# M.O.I.S.T. Concept for the Local Therapy of Chronic Wounds: An International Update

**DOI:** 10.1177/15347346241245159

**Published:** 2024-04-04

**Authors:** Joachim Dissemond, Paul Chadwick, Dot Weir, Paulo Alves, Kirsi Isoherranen, José Luis Lázaro Martínez, Terry Swanson, Andrea Gledhill, Matthew Malone

**Affiliations:** 1 Department of Dermatology, Venerology and Allergology, University of Essen, Essen, Germany; 2 1725 Birmingham City University, Birmingham, UK; 3 Saratoga Hospital Center for Wound Healing and Hyperbaric Medicine, Saratoga Springs, NY, USA; 4 59207 Universidade Católica Portuguesa, Institute of Health Sciences - Wounds Research Lab, Lisboa, Portugal; 5 Department of Dermatology and Allergology, University of Helsinki and Inflammation center, Helsinki University Hospital and Helsinki University, Helsinki, Finland; 6 Diabetic Foot Unit, Universidad Complutense de Madrid, Madrid, Spain; 7 Wound Education Research Consultancy, Warrnambool, Victoria, Australia; 8 Department of Podiatric Surgery, Trauma and Orthopaedics, Great Western Hospital NHSFT, Swindon, UK; 9 Research and Development, Molnlycke Healthcare AB, Gothenburg, Sweden; 10 Infectious Diseases Microbiology, School of Medicine, 6489 Western Sydney University, Sydney, Australia

**Keywords:** M.O.I.S.T. concept, wound healing, wound treatment, chronic wounds, local therapy

## Abstract

Chronic wounds remain a significant clinical challenge both for those affected and for healthcare systems. The treatment is often comprised and complex. All patients should receive wound care that is integrated into a holistic approach involving local management that addresses the underlying etiology and provides for gold standard therapy to support healing, avoid complications and be more cost effective. There have been significant advances in medicine over the last few decades. The development of new technologies and therapeutics for the local treatment of wounds is also constantly increasing. To help standardize clinical practice with regard to the multitude of wound products, the M.O.I.S.T. concept was developed by a multidisciplinary expert group. The M stands for moisture balance, O for oxygen balance, I for infection control, S for supporting strategies, and T for tissue management. Since the M.O.I.S.T. concept, which originated in the German-speaking countries, is now intended to provide healthcare professionals with an adapted instrument to be used in clinical practice, and a recent update to the concept has been undertaken by a group of interdisciplinary experts to align it with international standards. The M.O.I.S.T. concept can now be used internationally both as an educational tool and for the practical implementation of modern local treatment concepts for patients with chronic wounds and can also be used in routine clinical practice.

## Introduction

Chronic wounds substantially influence the quality of life of affected people and have a huge economic impact on health system budgets.^1^ Worldwide, about 1% of the adult population suffers from a chronic wound, which can have very aetiologies with estimates of significant increase due to an aging population.^2^ Most chronic wounds can be categorized as diabetic foot ulcers, venous leg ulcers, ischemic wounds due to peripheral arterial disease, or pressure inuries. Alongside the categorization of these wounds an understanding and appreciation of all other factors and rare wounds that can impair wound healing capability both intrinsic and extrinsic is important.^3,4^

The key to successful therapy of patients with chronic wounds is a combined approach of identifying and managing the cause of the wound, optimizing systemic disease processes and pathophysiology.^5^ The importance of correct diagnostics has been recently highlighted, as it is the basis of successful wound management.^6^ Additionally local wound therapy should always be provided to support wound healing and avoid complications.^7^ There are many products, therapies, and techniques for local wound treatment avaiable, so the individual choice can often be difficult. Meanwhile, it has also been scientifically proven that the use of advanced moist wound therapy supports the healing rates of chronic wounds.^8^

The choice of therapy and products is often based on the experience of the healthcare professionals (HCP), local guideleines and recommendations, and guidance from the evidence presented in scientific research. When scientific evidence is present this should be included in the decision-making process.^9^

## Local Wound Therapy From TIME to M.O.I.S.T

The diversity of options and changing landscape in wound care can be challenging for HCP to maintain oversight while maintaining a clinical role. Various concepts have been introduced to help structure the local therapy of chronic wounds. Internationally, the TIME concept has been the most widely adopted of these concepts.^10^ TIME was developed in 2003 by international experts and originally focused on the wound bed preparation. The acronym stands for Tissue, Inflammation or Infection, Moisture balance and Edges of wound or Epithelial advancement.^11^ Since this concept was introduced, there have been many advancements in therapies and emerging aspects of wound healing that cannot be represented directly in this concept.

The aim of the WundDACH, the umbrella organization of the German-speaking professional society in the field of wound treatment, was to evolve the proven TIME concept by including new therapy options for the local management of chronic wounds. In 2017, they published in German the first article of the M.O.I.S.T. concept,^12^ with a further update in 2022.^13^ In the development of M.O.I.S.T., the letters of the TIME concept described with T, I, and M were still considered to be contemporary and important and were therefore adopted in a slightly modified form for the M.O.I.S.T. concept. The letter E was originally used to describe factors about Epidermis^10^ and later Edge,^14^ that is, the advancement of the wound margin. In the M.O.I.S.T. concept, the letter E was not included because the group felt that these aspects, although important, were not directly part of local wound care. The nonulcerated skin of the wound edge and surrounding area must nevertheless be treated; however, no wound dressings are usually optomized for this. Instead of E, the WundDACH felt there was a greater importance of including the letters O for Oxygen balance and S for Supporting strategies to include new treatment options for targeted therapy in a much more differentiated way (Table 1).

**Table 1.** The Acronym M.O.I.S.T. Is Used to Describe a Modern Concept for the Local Therapy of Chronic Wounds.

• **M **= Moisture balance
• **O **= Oxyge balance
• **I **= Infection control
• **S **= Supporting strategies
• **T **= Tissue management

Since the M.O.I.S.T. concept originally came from the German-speaking countries and this took into account many national recommendations, it was now the plan of an interdisciplinary group of experts to revise and adapt this concept from an international point of view.

## Letters of the M.O.I.S.T. Concept

### M—Moisture Balance

The scientific work of George D. Winter in 1962 showed for the first time in a mouse model that a moist wound enviroment promoted wound healing.^15^ Until then, treating wounds with a dry approach was often the standard of care. Winter's findings led to a new paradigm shift in therapeutic concepts. However, at that time, appropriate products were still lacking to enable the therapy to be implemented well in practice. Primarily moist cotton compresses were used, which had to be moistened several times a day. In the following decades, the advantage of moist wound treatment was also confirmed in numerous clinical studies, so today this concept represents the gold standard of wound care for most chronic wounds.^16^

Providing local wound therapy is based on many different factors and should consider, among other things, the wound healing phases, exudate volumes and viscosities (Table 2). In moist wound therapy concepts, wounds should be neither too moist nor too dry.^17^ For example, dry wounds can be moistened with hydrogels. More often, however, there is an excess of exudate especially in venous leg ulcers and ulcers due to other causes of oedema. Therefore, wound products such as alginates, gelling fibers, or foams can be used. For larger amounts of exudate, superabsorbents or negative pressure wound therapies (NPWT) may be better suited. In addition to the selection of the primary wound dressing, it is often the secondary wound dressings that should absorb appropriate amounts of exudate.^18^

**Table 2.** Examples of Types of Wound Dressings Depending on the Amount of Exudate.

• Low: hydrogels, hydrocolloids, semipermeable films
• Moderate: foams, alginates, fibers
• High: foams, fibers, superabsorbers

### O—Oxygen Balance

The cells in the human body need a continuous supply oxygen for almost all metabolic processes necessary to support function and life. Since wound healing is accompanied by increased energy metabolism, the oxygen requirement here is even significantly higher compared to intact skin; in the case of infected wounds, this increases even further considerably.^19^ When oxygen is absent from tissue, it is termed hypoxic. Hypoxia occurs not only due to reduced blood flow in arterial disease patterns but also in almost all types of chronic wounds due to increased cellular activity/demands.^20^ If hypoxia persists, wound healing stagnates, and progressive tissue loss may occur in more severe cases. Normally, the partial pressure of oxygen (pO2) in healthy tissue should be about 100 mmHg. In contrast, values below 30 mmHg are often found in chronic wounds with stagnant wound healing. Transient hypoxia after tissue injury is initially physiological and promotes among other things angiogenesis and thus wound healing. However, prolonged hypoxia leads to stagnation of wound healing.^21^

With the development of new technologies including near-infrared spectroscopy, tissue oxygen tension, transcutaneous oxygen tension, or remission spectroscopy oxygen content in tissue can now be measured, therefore confirming if hypoxia is present.^22^ The current guidelines also recommend the measurement of skin perfusion pressure as a proven method for determining microcirculation. First the need for revascularization and accompanying aspects such as edema reduction through compression therapy should be clarified. However, these centrally important measures are not a direct component of the M.O.I.S.T. concept. This also applies to hyperbaric oxygen therapy (HBOT), in which the proportion of physically dissolved oxygen in the plasma when a patient breathes 100% oxygen while inside a treatment chamber at a pressure that is higher than sea-level pressure.^22^

To provide additional oxygen to chronic wounds, various therapeutic approaches for local therapy have also been developed in recent years (Table 3).^23^ All methods have in common that the oxygen is applied directly or indirectly to the wound surface, whereby different forms of application are used.

**Table 3.** Therapeutic Approaches With Oxygen for the Local Therapy of Chronic Wounds (Modified According to Gottrup et al.)^22^

• Continuous delivery of nonpressurized oxygen
• Low constant pressure oxygen in a contained chamber
• Higher cyclical pressure oxygen
• Oxygen release through dressing or gel
• Oxygen transfer

Small portable, battery-powered oxygen generators have been developed for the continuous delivery of nonpressurized oxygen, delivering a continuous stream of pure oxygen into occlusive dressings on wounds for 24 h a day.^24^ Low-pressure, constant-pressure devices consist of a simple plastic chamber or cuff that is placed around the wound. A constant pressure of up to 35 mmHg is then maintained in the chamber.^25^ Higher pressure oxygen devices combined with humidification apply oxygen locally at a pressure between 5 and 50 mmHg in a cyclic pressure waveform.^26^ When oxygen is delivered through a dressing or gel, it releases pure oxygen that is either embedded in a dressing or releases oxygen that is produced by a biochemical reaction in a hydrogel.^27^ For oxygen transfer, hemoglobin is used as an oxygen carrier in a wound spray. The mode of action is based on the physical effect of facilitated delivery in hypoxic areas.^28^

There is now increasing well-documented scientific evidence that local therapies with oxygen sustain healing, particularly in otherwise refractory chronic wounds.^29^ It is therefore not surprising that local oxygen therapy is one of the treatment methods recommended in the IWGDF guidelines for the treatment of people with DFU.^30^ Although the best data quality is available for patients with diabetic foot ulcers, it is now discussed that, in principle, almost all patients with chronic wounds can benefit from supplemental oxygen therapy.^23^

### Infection Control

The presence of microorganisms in wounds is physiological and does not always interfere with wound healing or contribute to causing infection. However, the clinical detection of wound infections can be challenging in patients in patients with chronic wounds. A failure to diagnose and properly manage wound infections can lead to serious consequences that may range from delayed wound healing, to spreading infection and sepsis.^31^ Diagnosis and interpretation of microbiological results are often difficult.^32^ For example, the validated Therapeutic Index for Local Infections (TILI) score is suitable for clinical diagnosis of local wound infections^33^ or the diabetic foot infection wound score.^34^ If systemic infection is suspected, serologic testing should be performed in addition to clinical signs and symptoms. To aid clinicians in the diagnosis and management of wound infections, several expert groups have provided guidelines and recommendations as to best practices.^35,36^

Infection control describes all antimicrobial strategies in the context of wound treatment. Topical antimicrobial wound therapy should be applied individually and specifically.^35^ Providing local delivery of topical antiseptics may yield several advantages over systemic approaches with the use of antibiotics. The latter remains the subject of much debate with current recommendations of the avoidance of using antibiotics as opposed to antiseptics topically for treating wound infections.^37^ As to clarify herein, the use of systemic antibiotics is not part of the “I” in M.O.I.S.T., as this covers the systematic approach for providing local wound therapy. As part of local wound infection control, clinicians need to understand the potential infectious wound microenvironment,^38^ which includes the types of bacteria responsible for causing the infection process, the type of infection (acute or chronic, local, or spreading) in addition to host factors such as immunocompromised.

One example to consider is wounds lacking clinical signs of infection but failing to respond to standard of care, which should raise suspcision of potential biofilm as a cause of chronic infection. Whether biofilm actually delays wound healing remains controversial.^39^ Nevertheless, targted antibiofilm strategies with proven efficacy in laboratory models, animal models and in human wounds could be employed. Additionally, there are various other topical antimicrobial (including antiseptics) substances available to clinicians for use against a wide variety of wound pathogens or microbial phenotypes (ie, planktonic, biofilm) that are recommended for use by expert opinion for the management of chronic wound infection/bioburden. Despite the heterogenity in proven efficacy of topical antimicrobials for biofilm good scientific evidence is available for traditional antispetics against planktonic bacteria such as octenidine (with phenoxyethanol), polihexanide, hypochlorus acid, and silver.^40,41^ The choice of type and mode of a topical antiseptic approach is also wide and usually includes antimicrobials that manage bacteria in the dressing and act as a barrier to microbial contamination. Delivery materials for such dressings include gelling fibers, ointments, pastes, gels, and novel systems such as chitosan, nanoparticle, and collagen matricies.^32^

Which specific format is best for the patient ultimately depends upon the wound microenvironment with factors such as high or low exudate levels, anatomical location, and space to be filled, all being examples of factors which may lead clincians to choose a specific topical antiseptic agent over another. More recently, nonmedicated dressings and devices have grown in popularity on the back of antimicrobial resistance fears. These dressings claim alternate mechanisms of antimicrobial action. Some examples include materials, which rely on physiochemical properties such as hydrophobic interactions with irreversible binding of bacteria to the dressing.^42^ Others report a hydroresponsive action causing passive wound exudate which contains bacteria from the wound to be drawn into the dressing with collateral bacterial trapping. An example for an innovative physical treatment approache is cold atmospheric plasma therapy.^43^

Regardless of the infection control strategy used, a critical review of the therapy should be performed on a regular basis. A suggested schedule is a review every 10 to 14 days. It should then be determined whether the treatment regimen should be continued or changed.^44^

### S—Supporting Strategies

There is an increasingly large heterogeneous group of wound products that are intended to actively intervene in the dysfunctional processes of wound healing (Table 4). The primary goal is then the targeted active influence of the wound environment. To be able to intervene specifically here, it is important to understand the various pathophysiological processes of wound healing with the corresponding biomarkers.^45,46^ However, much of this knowledge is currently based on basic science findings or studies from animal models. Although there are also some studies on this of humans with chronic wounds, reliable findings that also lead to targeted therapy based on individual diagnostics are largely lacking.^47,48^

**Table 4.** Examples of Therapeutic Points of Action that Are Already Used to Actively Support Wound Healing.

• Cytokines
• Growth factors
• Macrophages
• Matrix metalloproteinases
• pH values
• Reactive oxygen species
• Stem cells

Currently, the most widely used approach in this wound therapy sector is the therapeutic interaction with matrix metalloproteinases (MMPs) because of their imbalance in the microenvironment of chronic wound.^49^ Matrix metalloproteinases are a group of zinc-dependent endopeptidases that catalyze the cleavage of peptide bonds. The physiological counterparts are the tissue inhibitors of metalloproteinases (TIMPs). The centrally important role of MMPs in physiological wound healing has been described primarily in the context of the degradation of extracellular matrix proteins.^50^ Matrix metalloproteinases also have numerous other functions in the regulation of physiological and pathological processes. They are important for angiogenesis, tumor growth, and as signaling molecules, among others. It has been shown that in most chronic wounds there is an impaired ratio of MMPs and TIMPS. This excess of MMPs is the attack point of various wound products that include, for example, collagen and cellulose, polyhydrated ionogens-5, or sucrose octasulfate.^51,52^ Good evidence with multiple RCTs exists for the potassium salt of sucrose octasulfate.^53,54^

In other therapeutic approaches, growth factors are used to support wound healing in chronic wounds. They regulate a wide variety of intracellular processes and play a particularly important role in tissue development. In the context of wound healing, the effects of growth factors in angiogenesis have been studied extensively. Growth factors currently used for wound treatment are for example platelet-derived growth factor,^55^ granulocyte-macrophage colony-stimulating factor,^56^ and epidermal growth factor.^57^ In addition, there are several procedures in which fractions with growth factors are obtained from the patient's blood for wound treatment.^58^ Other biological products such as amniotic membrane, adipose-derived,^59^ or mesenchymal stem cells^60^ also be used. In this context, other skin substitute products for wound treatment can be mentioned.^61^

It has been known for a long time that the pH value influences all processes in the human body. In this respect, it is obvious to actively influence the pH value in wound healing. The current findings on the pH values in wounds are very much dependent on the measurement methods used and many other factors are and can be influenced.^62^ Every wound product has a pH value and can therefore potentially actively influence the wound milieu. Only a few wound products have the pH value specified by the manufacturers. In addition, today there is also a lack of precise knowledge about when which wounds benefit from which pH values, so currently this targeted wound treatment cannot be used.^63^ Other very interesting therapeutic approaches to actively support the healing process include local therapies with negatively charged microspheres,^64^ chitosan,^65^ coagulation factors,^66^ or extracellular matrix proteins.^67^

Even if some measurement instruments for the wound environment have already been developed,^68^ it is currently still not possible to reliably measure the multitude of potentially influential aspects of the wound environment in clinical practice. In addition, there is a lack of meaningful studies demonstrating when values should be actively changed and what constitutes an optimal wound environment. In this respect, the use of these certainly very interesting wound healing products is mostly carried out in otherwise therapy-refractory wounds according to the trial-and-error principle. This will certainly change in the future as soon as appropriate measurement methods are available. Here, it is wearables or smart dressings that measure individual factors in real time and then, if necessary, treat them specifically.^69^

### T—Tissue Management

Tissue management describes all measures of local wound therapy that contribute to wound bed preparation. The first step usually involves measures of wound cleansing and debridement.^70^ Wound cleansing or wound irrigation means the removal of nonadherent components with solutions. Although the scientific evidence of this wound cleansing has not yet been conclusively demonstrated,^71^ it is part of the gold standard of modern wound treatment concepts in patients with chronic wounds.^31^ In addition to residual dressings, it is often dried wound exudates that are removed as atraumatically as possible. Sterile or preserved solutions should be used and adapted to the respective national or local specifications.^18^

In chronic wounds, debridement describes the removal of adherent, dead tissue, crusts, or foreign bodies (Table 5). The main goals are to prevent and combat wound infections, biofilm formation, improve the assessability of the wound, and promote wound healing.^72^ Surgical debridement refers to the complete removal of devitalized tissue down to intact tissue structures. This usually involves tissue and vascular injury with bleeding.^73^ Various forms of therapy are summarized as mechanical debridement, in which loosely adherent wound components are removed from the wounds as far as possible atraumatically, for example with cotton compresses or monofilament-fiber pads. Biosurgery is the treatment of wounds with sterile cultured fly maggots. During autolytic debridement, the body's own proteolytic enzymes are released, and phagocytes are activated among other things. Autolytic debridement is facilitated by the creation of a moist wound healing environment. For proteolytic debridement, products containing bromelain, collagenase, or streptokinase and streptodornase, which hydrolyze peptide bonds, are most commonly used. The group of technical debridements includes, for example, hydrosurgery, lasers, or ultrasound devices.^72^

**Table 5.** Options for Debridement in Chronic Wounds (Modified According to Strohal et al.)^72^

• Autolytic debridement
• Biosurgical debridement
• Mechanical debridement
• Osmotic debridement
• Proteolytic/enzymatic debridement
• Surgical/sharp debridement
• Technical debridement

For further wound treatment, neutral wound dressings, or physical devices such as NPWT can subsequently be used. Negative pressure wound therapy is an active wound management system that utilizes controlled negative (subatmospheric) pressure, which is applied uniformly to the wound through an open cell foam or other interface dressing in a continuous or intermittent fashion. There is now good evidence available for this wound treatment method in improving wound healing in various types of chronic wounds.^74^

Other physical treatment options that can currently be used for wound treatment include for example electrostimulation, extracorporeal shock waves, concurrent optical and magnetic stimulation, low-intensity laser therapy, or water-filtered infrared A-light.^75^

## Discussion

**Figure 1.**
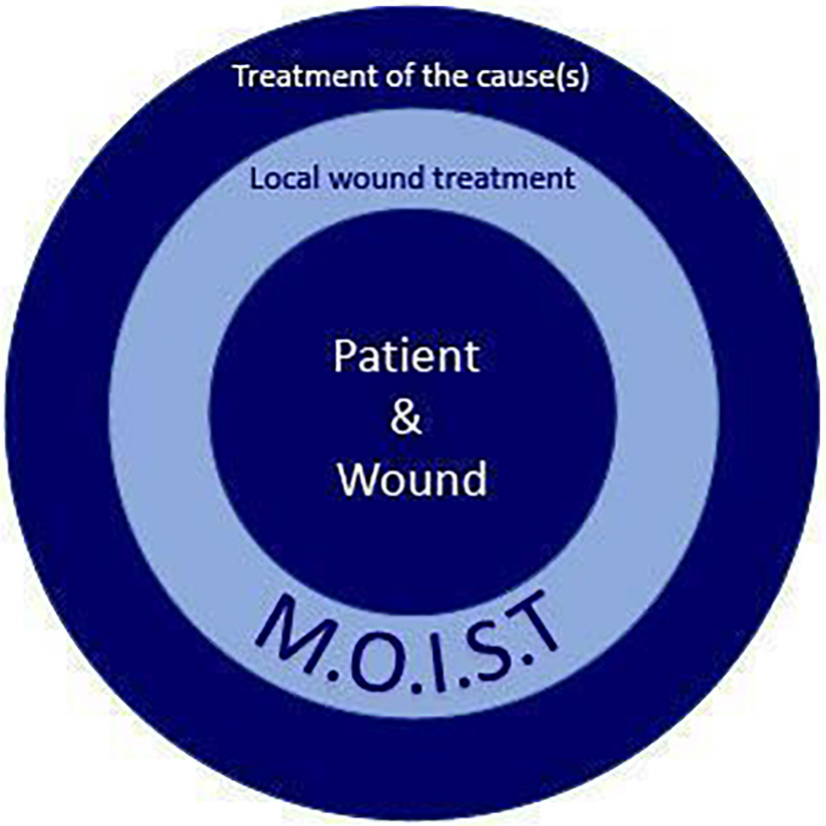
The patient is always at the center of holistic wound treatment. The M.O.I.S.T. concept then describes the local treatment of wounds. In addition, however, it is essential to treat the underlying cause(s). Successful wound treatment can only be achieved through the coordinated interaction of these different components.

The basis of successful wound management relies in accurate diagnostics and after that comes the decision of local therapy (Figure 1). When using the M.O.I.S.T. concept, it is important to keep in mind that not all letters need to be considered for all patients. Rather, it is a matter of at least considering each of the aspects and then considering the applicability based on a through wound assessment. For example, treating all chronic wounds with antimicrobials is unnecessary. However, an appropriate assessment of the presumed bioburden should always be made. Furthermore, it should be noted that the implementation of therapy according to the letters of the M.O.I.S.T. concept does not have to be done successively. Therefore, it should be understood as an overall concept. For example, in many patients, it may make sense to start wound treatment with T, that is, debridement. It is also clear that the effects of the individual treatment methods are not always clearly attributable to one letter. As an example, NPWT, which is usually used primarily for T, can also support M and be used in combination with an antimicrobial instillation for I. However, this should generally be seen as advantageous, as treatment concepts then need to be planned in a less complex manner.

When making the final decision on which wound care products to use for which patients individual and economic factors should always be considered. It is very important to consider the individual needs and capabilities of patients in a shared decision-making process.^76^ In addition, products must also be available and fit the patient's particular form of care. This is particularly important when self-management is planned and supported with education.

## Conclusion

Treatment concepts for patients with chronic wounds should always include adequate local wound therapy based on accurate diagnostics. Local wound therapy cannot replace causative treatment strategies, but it will support wound healing and prevent complications if integrated into a comprehensive overall management plan. To better structure the multitude of wound products, the M.O.I.S.T. concept was developed. This concept offers HCP support for systematic planning and education with regard to the local therapy of patients with chronic wounds.
